# Consensus guidelines for management of hyperammonaemia in paediatric patients receiving continuous kidney replacement therapy

**DOI:** 10.1038/s41581-020-0267-8

**Published:** 2020-04-08

**Authors:** Rupesh Raina, Jirair K. Bedoyan, Uta Lichter-Konecki, Philippe Jouvet, Stefano Picca, Nicholas Ah Mew, Marcel C. Machado, Ronith Chakraborty, Meghana Vemuganti, Manpreet K. Grewal, Timothy Bunchman, Sidharth Kumar Sethi, Vinod Krishnappa, Mignon McCulloch, Khalid Alhasan, Arvind Bagga, Rajit K. Basu, Franz Schaefer, Guido Filler, Bradley A. Warady

**Affiliations:** 1 Department of Nephrology, Akron Children’s Hospital, Akron, OH USA; 2 Akron Nephrology Associates/Cleveland Clinic Akron General, Akron, OH USA; 3 Center for Human Genetics, University Hospitals Cleveland Medical Center and Department of Genetics and Genome Sciences, Case Western Reserve University, Cleveland, OH USA; 4 Division of Medical Genetics, UPMC Children’s Hospital of Pittsburgh, Pittsburgh, PA USA; 5 Department of Paediatrics, Sainte-Justine Hospital, University of Montreal, Montreal, Quebec Canada; 6 Division of Nephrology and Dialysis, Department of Paediatrics, Bambino Gesù Children’s Hospital and Research Institute, Rome, Italy; 7 Children’s National Rare Disease Institute, The George Washington University, Washington, DC USA; 8 Department of Emergency Medicine, University of São Paulo School of Medicine, São Paulo, Brazil; 9 Department of Pediatrics, Rainbow Babies and Children’s Hospital, Cleveland, OH USA; 10 Department of Pediatric Nephrology, Children’s Hospital of Michigan, Detroit, MI USA; 11 Pediatric Nephrology & Transplantation, Children’s Hospital of Richmond, Virginia Commonwealth University, Richmond, VA USA; 12 Paediatric Nephrology & Paediatric Kidney Transplantation, Kidney and Urology Institute, Medanta, The Medicity Hospital, Gurgaon, India; 13 Northeast Ohio Medical University, Rootstown, OH USA; 14 Red Cross War Memorial Children’s Hospital, University of Cape Town, Cape Town, South Africa; 15 Department of Paediatrics, King Saud University, College of Medicine, Riyadh, Saudi Arabia; 16 Division of Paediatric Nephrology, All India Institute of Medical Sciences, New Delhi, India; 17 Department of Pediatric Critical Care Medicine, Children’s Healthcare of Atlanta, Atlanta, GA USA; 18 Division of Paediatric Nephrology, University Children’s Hospital Heidelberg, Heidelberg, Germany; 19 Division of Paediatric Nephrology, Department of Paediatrics, Western University, London, Ontario Canada; 20 Division of Nephrology, University of Missouri–Kansas City School of Medicine, Children’s Mercy, Kansas City, MO USA

## Abstract

Hyperammonaemia in children can lead to grave consequences in the form of cerebral oedema, severe neurological impairment and even death. In infants and children, common causes of hyperammonaemia include urea cycle disorders or organic acidaemias. Few studies have assessed the role of extracorporeal therapies in the management of hyperammonaemia in neonates and children. Moreover, consensus guidelines are lacking for the use of non-kidney replacement therapy (NKRT) and kidney replacement therapies (KRTs, including peritoneal dialysis, continuous KRT, haemodialysis and hybrid therapy) to manage hyperammonaemia in neonates and children. Prompt treatment with KRT and/or NKRT, the choice of which depends on the ammonia concentrations and presenting symptoms of the patient, is crucial. This expert Consensus Statement presents recommendations for the management of hyperammonaemia requiring KRT in paediatric populations. Additional studies are required to strengthen these recommendations.

## Abstract (web summary)

This expert Consensus Statement from the Pediatric Continuous Renal Replacement Therapy (PCRRT) workgroup presents recommendations for the management of hyperammonaemia requiring kidney replacement therapy in paediatric populations. Additional studies are needed to strengthen these recommendations, which will be reviewed every 2 years.

## Introduction

In aqueous solution, ammonia exists as ammonium hydroxide ions, which aid in maintaining acid–base homeostasis^1,2^, as expressed by the following equilibrium:

$${{\rm{N}}{\rm{H}}}_{3}\,+\,{{\rm{H}}}_{2}{\rm{O}}\rightleftarrows ({{{\rm{N}}{\rm{H}}}_{4}}^{+})\,+\,({{\rm{O}}{\rm{H}}}^{-})$$

Hyperammonaemia is the excessive accumulation of ammonia in the blood, which can result in moderate to severe neurological impairment and cerebral oedema. Ammonia is produced by amino acid catabolism, the activity of glutamine dehydrogenase in the liver, kidney, pancreas and brain, and by the deamination of AMP during exercise in skeletal muscle^1^. Most ammonia produced enters the urea cycle in hepatocytes, is excreted in the urine as urea or is converted into glutamine, a fraction of which is also excreted by the kidneys^1,3^.

**Fig. 1:**
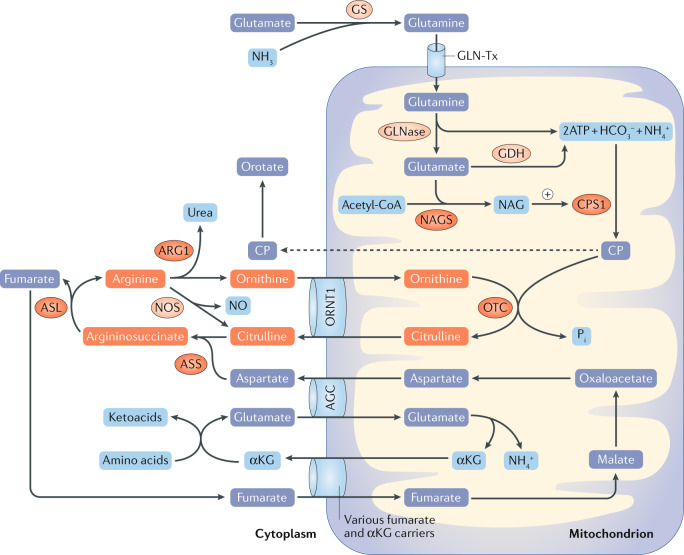
Urea cycle dysfunction results in toxic accumulation of ammonia in the blood. Hyperammonaemia in neonates and infants is typically caused by inborn errors of metabolism. Primary urea cycle disorders are caused by congenital deficiency of any of the six urea cycle enzymes: *N*-acetylglutamate synthase (NAGS), carbamoyl phosphate synthase I (CPS1), ornithine transcarbamylase (OTC), argininosuccinate synthetase (ASS), argininosuccinate lyase (ASL) and arginase 1 (ARG1). These deficiencies result in severe hyperammonaemia and the accumulation of both urea and the substrate of the missing or defective enzyme. For example, the accumulation of carbamoyl phosphate (CP; dashed line) results in greatly increased production and excretion of orotate. Secondary inhibition of the urea cycle is caused by abnormalities that reduce the activity of other enzymes involved in amino acid processing. These deficiencies cause organic acidaemias, such as methylmalonic acidaemia, propionic acidaemia, isovaleric acidaemia and multiple carboxylase deficiency, as well as (typically mild to moderate) hyperammonaemia. Hyperammonaemia can also occur following exposure to drugs, such as valproic acid. Finally, paediatric patients with liver diseases or acute kidney injury are also susceptible to hyperammonaemia owing to impaired metabolism or excretion of urea, respectively^68^. αKG, α-ketoglutarate; AGC, aspartate–glutamate carrier; GDH, glutamate dehydrogenase; GLNase, glutaminase; GLN-Tx, glutamine transporter; GS, glutamine synthase; NAG, *N*-acetylglutamate; NOS, nitric oxide synthase; NO, nitric oxide; ORNT1, ornithine translocase.

Typically, blood ammonia concentrations ≤35 µmol/l (<60 µg/dl) are considered normal, whereas high concentrations can produce toxic effects and levels >200 µmol/l (341 µg/dl) are associated with poor neurological outcomes^2^. The primary differential diagnosis of hyperammonaemia in infants and children relates to inborn errors of metabolism, including urea cycle disorders (UCDs) and organic acidaemias^2^. Toxic levels of ammonia can be due to either a primary or a secondary deficiency of the urea cycle. Congenital deficiency of any of the six enzymes within this cycle — *N*-acetylglutamate synthase (NAGS), carbamoyl phosphate synthase I (CPS), ornithine transcarbamylase (OTC), argininosuccinate synthetase (ASS), argininosuccinate lyase (ASL) and arginase 1 — will result in a build-up of both ammonia and the substrate of the specific enzyme^4^. UCDs occur in ~1 in 35,000 births, among which OTC deficiency is the most common, with an incidence of 1 in 56,500 births^4^. Hyperammonaemia due to secondary inhibition of the urea cycle occurs in the context of other congenital metabolic abnormalities (such as organic acidaemias), following exposure to certain drugs (such as valproic acid) and in various liver diseases. Organic acidaemias, including methylmalonic acidaemia, propionic acidaemia, isovaleric acidaemia and multiple carboxylase deficiency occur in ~1 in 21,000 births and typically lead to mild to moderate hyperammonaemia due to competitive inhibition of NAGS^5–9^. Paediatric patients with acute liver or kidney injury are also susceptible to an accumulation of ammonia due to impaired metabolism and excretion processes, respectively^2^ (Fig. 1).

Hyperammonaemia is defined as >100 µmol/l (170 µg/dl) in neonates or $\ge$50 µmol/l (85 µg/dl) in term infants, children and adolescents^10^. Individual laboratory reference intervals vary and some age dependency of ammonia levels is evident (that is, levels are higher in premature neonates). Early onset hyperammonaemia presents in neonates within the first few days of life after they start to feed and can no longer rely on maternal (placental) transport to appropriately eliminate the accumulating ammonia^2,4,7^. Symptoms in preterm neonates typically result from transient hyperammonaemia of the newborn, which is characterized by the absence of organic acidurias and normal activity of urea cycle enzymes. This subtype is usually associated with complete recovery, usually without needing treatment.

**Fig. 2:**
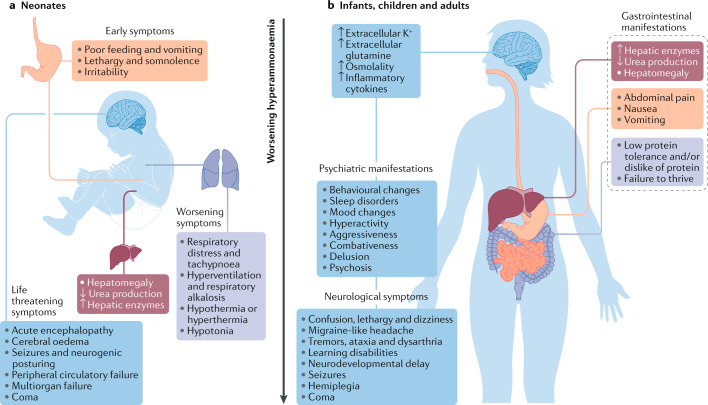
Clinical manifestations of hyperammonaemia. The clinical features of an acute hyperammonaemic episode are influenced by the age of the patient and the underlying cause of hyperammonaemia — that is, the specific urea cycle enzyme deficiency or organic acidaemia. **a** | In neonates, early symptoms include lethargy, loss of appetite and vomiting. If ammonia levels continue to rise, symptoms progress to hypotonia and hyperventilation resulting in respiratory alkalosis. Severe hyperammonaemia is characterized by an acute encephalopathy, seizures, and, if untreated, coma and death. **b** | Late-onset hyperammonaemia (that is, in infants, children and adults) typically results from a partial or mild urea cycle enzyme deficiency exacerbated by exposure to stressors such as drug treatment. These individuals typically present with failure to thrive and abdominal symptoms, accompanied by psychiatric manifestations such as irritability, learning disabilities, delusion and psychosis. Patients might additionally have neurological symptoms, including neurodevelopmental delay, seizures and hemiplegia. The psychiatric and neurological manifestations of hyperammonaemia are attributable to increased brain levels of ammonia, which is metabolized to glutamine by astrocytes. The resulting high extracellular levels of potassium and glutamine cause increased intracellular osmolality and cerebral oedema, leading to neuronal damage and the release of inflammatory cytokines.

In hyperammonaemia, the acute rise in ammonia levels in the brain leads to increased levels of extracellular potassium and metabolism of ammonia to glutamine by astrocytes^1,3,11^. These changes result in increased intracellular osmolality, cerebral oedema and the release of inflammatory cytokines. High levels of extracellular potassium and glutamate released by astrocytes lead to neuronal damage^1,3^. Elevated levels of glutamine, which is the end product of ammonia detoxification, is a key factor in both hepatic encephalopathy and ammonia-related neurotoxicity^12^. Apart from hyperammonaemia, adverse effects on the nitric oxide pathway are also thought to lead to brain damage in patients with UCDs^13^. The clinical abnormalities observed vary for each specific UCD, but most patients tend to have similar symptoms^14^. The clinical features of an acute hyperammonaemic episode depend on the age of the patient and the cause of the elevation in plasma ammonia levels. Early symptoms include lethargy, loss of appetite and vomiting. As ammonia levels rise, symptoms can progress to hyperventilation resulting in respiratory alkalosis, hypotonia, ataxia, disorientation, seizures and, if untreated, coma and death^15^. Late-onset hyperammonaemia in children, adolescents or adults can result from a partial or mild deficiency in a urea cycle enzyme that is exacerbated by certain stressors. Such children or adolescents present with failure to thrive, irritability, seizures, vomiting, ataxia and intellectual disabilities. Management of hyperammonaemia is challenging in paediatric populations given the non-specific clinical symptoms, the age-specific aetiologies and the lack of consensus in the treatment plan. Historically, the prognosis in neonates with hyperammonaemia was poor, but current treatments, along with prompt identification of hyperammonaemia, have considerably improved the survival of affected neonates^16^ (Fig. 2), although updated survival data are not available.

This Consensus Statement presents guidelines for non-kidney replacement therapy (NKRT) and kidney replacement therapy (KRT) of hyperammonaemia in paediatric patients. KRT includes peritoneal dialysis (PD), haemodialysis (HD) and continuous kidney replacement therapy (CKRT). Varieties of CKRT include continuous venovenous haemofiltration (CVVH), continuous venovenous haemodiafiltration (CVVHDF) and hybrid therapy. These guidelines and the included recommendations will be reviewed every 2 years.

## Methods

### The PCRRT workgroup

The PCRRT workgroup comprises an international expert panel of paediatric nephrologists from various professional societies representing a diverse paediatric population. The workgroup chair was T.B. (paediatric nephrologist, chair and founder of PCRRT). Co-chairs were J.K.B. (clinical and biochemical geneticist, Department of Genetics and Genome Sciences, Associate Director of the Center for Human Genetics (Metabolism Section) and Section Medical Director, CIDEM Laboratory, University Hospitals Cleveland Medical Center) and R.R. (adult and paediatric nephrologist). The workgroup guidelines were discussed with international panellists at the Pediatric Critical Care Nephrology & Renal Replacement Therapy workshop (sponsored by PCRRT) on 11 April 2019. These experts met to discuss and develop recommendations for the management of paediatric hyperammonaemia with KRT. Further meetings were held at a consensus conference during the International Society of Nephrology (ISN) biennial World Congress of Nephrology in Melbourne, Australia, 12–15 April 2019. The workgroup members all disclosed any conflicts of interest, and all participated in a careful review of the objectives, background, rationale and statistical methods used in this study.

The literature search, article review, data extraction and results were completed by the PCRRT co-chairs, who submitted their results to the workgroup for discussion. All individuals were responsible for reviewing and proposing recommendations for KRT in children with hyperammonaemia. Disagreements between panel members were resolved by quantifying votes using the RAND Corporation/University of California Los Angeles (UCLA) appropriateness method and subsequently calculating a disagreement index^17,18^. The modified Delphi method was used to establish the strength of each recommendation. Grading of recommendations, assessments, development, and evaluation (GRADE) criteria^19^ were used to establish the evidence level for each clinical recommendation.

### Data selection and review

The PubMed/MEDLINE, EMBASE and Cochrane databases were searched to identify publications relevant to hyperammonaemia and KRT in the paediatric population. Medical subject headings (MeSH terms) used in the creation of the search strategy included “renal replacement therapy”, “renal dialysis*”, “kidney replacement”, “hemofiltration*”, “hemodialysis”, “dialysis”, “peritoneal dialysis”, “CAPD”, “hemodiafiltration*”, “hemoperfusion*” and “hyperammonemia*”. Asterisks denote terms that were expanded to search all related terms on the familial hierarchy. The MeSH term “hyperammonemia” included narrower terms such as “urea cycle disorder”, “hyperlysinemia”, “hyperornithinemia”, “hyperammonemia and homocitrullinuria (HHH) syndrome”, “methylmalonic aciduria”, “CPS deficiency disease”, “OTC deficiency disease” and “propionic aciduria”. The search strategy was limited to patients aged 0–18 years. Supplementary Box 1 lists the full and specific search strategies used.

The initial database searches returned a total of 477 citations. All citations obtained were reviewed by two independent reviewers. A PICO (patient, problem or population; intervention; comparison, control or comparator; outcomes) model table was constructed to illustrate the inclusion and exclusion criteria (Table 1). Each title, abstract and full-text article was assessed by two independent reviewers. To settle disputes and assess inter-rater agreements, a third independent reviewer also assessed the articles. After removal of duplicates, 329 citations remained, of which 118 were considered for full-text review. Supplementary Table 1 shows the PRISMA statement checklist that was used in reporting the selected articles^20^. The Cochrane risk of bias assessment tool for randomized control trials and the Newcastle–Ottawa scales for cohort studies were used to assess the quality of the included studies^21,22^.

**Table 1:** Study eligibility criteria

Category	Inclusion criteria	Exclusion criteria
Population	Paediatric patients (aged 0–18 years) who received non-kidney replacement therapy or kidney replacement therapy	Adult patients (aged >18 years)
Outcomes analysed	Hyperammonaemia	NA
Study type	Case reports, retrospective studies	Meta-analyses, systemic reviews, abstracts

NA, not applicable

The workgroup considered data from 28 of these studies to be most relevant for formulating the guidelines. A total of 172 patients were included in these 28 studies, with ages ranging from 1 day to 7 years. Hyperammonaemia was associated with a multitude of aetiologies, the most common of which were inborn errors of metabolism. The most frequent inborn errors of metabolism were CPS deficiency (*n* = 16), propionic acidaemia (*n* = 15) and methylmalonic acidaemia (*n* = 13). Indications for HD included hyperammonaemia refractory to medical management and hyperammonaemic coma. Illness severity ranged from haemodynamically stable to hyperammonaemic coma. Dialysis duration ranged from 2 h to 19 days. The success rates for each modality were 65% for PD (*n* = 23), 100% for intermittent HD (*n* = 5), 60% for CKRT (*n* = 92), and 100% for extracorporeal membrane oxygenation (ECMO) combined with CKRT (*n* = 18). Criteria for success consisted of a decrease in ammonia to normal levels and a good clinical outcome with minimal rebound hyperammonaemia. The most pertinent data on patient characteristics, HD parameters and outcomes are summarized in Table 2 (an expanded table is provided as Supplementary Table 2).

**Table 2:** Summary of data from the most important included studies

Study population	KRT duration	Ammonia levels (clearance)	Ref.
***Peritoneal dialysis***			
9 patients, mean age 11.7 ( ± 9.7) days, with MSUD (*n* = 3), hyperammonaemia and/or organic acidaemias (*n* = 4) or UCDs (*n* = 2)	4.6 ± 1.9 days	Pre-KRT peak range 531–1,533 μmol/l (904.35–2,610.85 μg/dl); post-KRT 300 ± 440 μmol/l (510.93 ± 749.36 μg/dl) (66%)	^37^
7 patients, mean age 2–3 days, with CPS deficiency (*n* = 1), PA (*n* = 3), MMA (*n* = 1), OTC deficiency (*n* = 1) or ASL (*n* = 1)	Mean 2.4 days (range 5 h to 15 days)	Pre-KRT >1,000 μmol/dl (1,703.1 μg/dl); 20 h after KRT <200 mg/dl (340.62 μg/dl) (57%)	^56^
***Haemodialysis***			
1 patient aged 2 days with citrullinaemia	2 rounds: 4.5 h and 2 h	Pre-KRT peak 874 μmol/l (1,488.51 μg/dl); post-KRT 588 μmol/l (1,001.42 μg/dl), then 89 μmol/l (151.58 μg/dl) (100%)	^48^
4 patients aged 7 h to 10 days with transient hyperammonaemia (*n* = 2), MMA (*n* = 1) or OTC deficiency (*n* = 1)	Mean 4–5 h; 2 patients required multiple sessions	Pre-KRT average 595 μmol/l (1,013.34 μg/dl); post-KRT 180 μmol/l (306.56 μg/dl) (100%)	^46^
1 patient aged 2 years with CPS deficiency	Mean 14 h	Pre-KRT peak hyperammonaemia 765 μmol/l (1,302.87 μg/dl); post-KRT 153 μmol/l (260.57 μg/dl) (100%)	^57^
1 patient aged 3 days with MMA	2 h HD then 14 h HF	Pre-KRT peak hyperammonaemia 1,533 μmol/l (2,610.85 μg/dl); post-KRT 209 μmol/l (292.76 μg/dl) (100%)	^31^
***Continuous kidney replacement therapy***			
14 patients, mean age 5.5 ± 7.4 months, with OTC deficiency (*n* = 5), MSUD (*n* = 4) or CPS deficiency (*n* = 5)	CVVHDF (*n* = 11) or CVVHD (*n* = 3) mean 16.6 ± 15.6 h	(*n* = 10) Post-KRT <200 μmol/l (340.62 μg/dl) (86.70%)	^58^
21 patients, mean age 4.1 ± 2.4 days (*n* = 19), 1 year (*n* = 1), 7 years (*n* = 1), with citrullinaemia (*n* = 8), OTC deficiency (*n* = 3), MMA (*n* = 2), CPS deficiency (*n* = 3), PA (*n* = 1), glutaric acidaemia type II (*n* = 1), argininaemia (*n* = 1), unknown diagnosis (*n* = 2)	CVVHD mean 42 ± 30.4 h (*n* = 17); PD mean 59.4 ± 87.2 h (*n* = 4)	Pre-KRT peak hyperammonaemia 1,225.8 ± 1,172.9 μmol/l (1,717 ± 1,643 μg/dl); 50% reduction within 4.7 ± 2.5 h for CVVHD (84%) and within 13.5 ± 6.2 h for PD (50%)	^59^
3 patients aged 3–7 days with MMA	CAVHDF mean 19 h	Pre-KRT peak hyperammonaemia 449–932 μg/dl; post-KRT 85–124 μg/dl (100%)	^60^
21 patients aged 15.7 ± 11.7 days with anuria and hyperhydration (*n* = 17), azotaemia with anuria (*n* = 1), HUS (*n* = 1) or neonatal hyperammonaemia (*n* = 2)	CVVH (*n* = 12); CVVHD (*n* = 1); CAVHF (*n* = 1); CVVHDF (*n* = 6); mean 66.8 ± 56.6 h	Pre-KRT peak >1,300 μmol/l (2,214.03 μg/dl); post KRT 347 μmol/l (590.98 μg/dl) with CVVHD; 104 μmol/l (177.12 μg/dl) with CVVHDF (42.90%)	^61^
4 patients aged 2–3 days with CPS deficiency (*n* = 1), MMA (*n* = 1) or pyruvate carboxylase deficiency (*n* = 2)	CVVHDF mean 30 h	Pre-KRT 152.66–1,051.02 μmol/l (260–1,790 μg/dl; post-KRT <117.43 μmol/l (200 μg/dl) (50%)	^39^
12 patients aged 4 days to 2 months with MSUD (*n* = 4), PA (*n* = 3), CPS deficiency (*n* = 2), CPS deficiency (*n* = 2), ASL deficiency (*n* = 1)	CVVHD mean 29.5 h (*n* = 7), PD dwell time 30–60 min; mean 73 h (*n* = 5)	Pre-KRT peak 3,820 μmol/l (6,505.84 μg/dl); 50% ammonia reduction time 7.1 h for CVVHD and 17.9 h for PD (83%)	^38^
***Hybrid therapy***			
21 patients aged 56.2 ± 71.0 months with UCDs (*n* = 14), organic acidaemias (*n* = 5), idiopathic hyperammonaemia (*n* = 1) or Reye syndrome (*n* = 1)	HD then CVVHD mean 6.1 ± 9.8 days	Pre-KRT 721.4 ± 467.2 μmol/l (1,010.5 ± 654.4 μg/dl); post-KRT <200 μmol/l (340.62 μg/dl) (100%)	^49^
2 patients aged 5 days with PA	CVVHD with HF–ECMO; 70–80 ml/h, 40–90 ml/h; 33 h	Pre-KRT >968 μmol/l (1,355.90 μg/dl); post-KRT 102 μmol/l (142.9 μg/dl) (100%)	^50^
2 patients aged 5 days and 4 days with ASL deficiency (*n* = 10 or UCD (*n* = 1)	HD–ECMO 2 h	Pre-KRT 780 μmol/l (1,328.42 μg/dl), 1,500 μmol/l (2,554.65 μg/dl); post-KRT 24 μmol/l (40.88 μg/dl), 35 μmol/l (59.61 μg/dl), respectively (100%)	^51^
13 patients, mean age 38.1 weeks (IQR 37.0–39.0 weeks), with deficiency of ASL (*n* = 6), CPS (*n* = 5), OTC (*n* = 2), isovaleric acid CoA dehydrogenase (*n* = 1) propionyl CoA carboxylase (*n* = 1) or organic acidaemia (*n* = 2)	HD–ECMO 7.3 h (IQR 3.6–13.5 h)	Pre-KRT peak 1,041 μmol/l (1,772.93 μg/dl); post-KRT <200 μmol/l (280.1 μg/dl) (100%)	^52^

Expanded information on the included studies is available online in Supplementary Table 2 (refs^31,37–39,46,48–53,56–67^). ASL, argininosuccinate lyase; CAVHDF, continuous arteriovenous haemodiafiltration; CAVHF, continuous arteriovenous haemofiltration; CoA, coenzyme A; CPS, carbamoyl phosphate synthetase; CVVH, continuous venovenous haemofiltration; CVVHD, continuous venovenous haemodialysis; CVVHDF, continuous venovenous haemodiafiltration; ECMO, extracorporeal membrane oxygenation; HD, haemodialysis; HF, haemofiltration; HUS, haemolytic uraemic syndrome; IQR, interquartile range; KRT, kidney replacement therapy; MMA, methyl malonic acidaemia; MSUD, maple syrup urine disease; OTC, ornithine transcarbamylase; PA, propionic acidaemia; PD, peritoneal dialysis; UCD, urea cycle disorders.

In the literature review, very few randomized controlled trials and meta-analyses were identified, and those identified were excluded from the scope of the present expert Consensus Statement on the basis of insufficient quality and a lack of pertinent information. All the recommendations were made on the basis of case reports and retrospective studies (Table 3). Clearly, high grades of evidence^19^ could not be provided based on case reports and retrospective studies describing a few, rare and scattered paediatric patients with hyperammonaemia requiring dialysis. Yet, guidelines with low evidence grading can still be of immense value to clinicians while waiting for additional evidence. Another limitation of the study concerns the lack of consensus on the preferred modality for KRT, the type of dialyser, membrane and filtration rate; these parameters were all institution-dependent and thus not generalizable. Indications for KRT were also variable; some studies initiated KRT at a certain ammonia level (typically >400 μmol/l (681 μg/dl)), whereas others initiated KRT when the patient was haemodynamically unstable, irrespective of the patient’s ammonia level. Future large-scale studies are necessary to establish more specific guidelines for KRT in paediatric patients with hyperammonaemia.

**Table 3:** Dialysis ammonia clearance and filtration fractions

Number of patients	Dialysis modality	Qb (ml/min)	Qd (ml/min)	Ammonia clearance (ml/min/kg body weight)	Ammonia filtration fraction (%)
3	CAVHD	10–20	8.3 (0.5 l/h)	0.87–0.97	12.5–14.3
3	CVVHD	20–40	33.3–83.3 (2–5 l/h)	2.65–6.80	53.0–58.0
2	HD	10–15	500	3.95–5.37	95.0–96.0

CAVHD, continuous arteriovenous haemodialysis; CVVHD, continuous venovenous haemodialysis; HD, haemodialysis; Qb, blood flow rate; Qd, dialysis fluid flow rate. Based on data from ref.^34^.

## Consensus panel recommendations

Prompt identification and treatment of hyperammonaemia are imperative to optimize the outcome of a hyperammonaemic crisis and to avoid irreversible brain damage.

### Initial medical management guidelines

In the initial medical management of acute hyperammonaemia, the patient should be immediately stabilized (circulation, airway and breathing), vital signs should be addressed, blood glucose levels monitored, intravenous access should be established, airways should be maintained by intubation and ventilation if necessary, and adequate rehydration should be started, typically using a dextrose-containing fluid at a high infusion rate^23^. In patients in hyperammonaemic crisis, laboratory testing is required to establish the cause of hyperammonaemia^24^. Accurate measurement of plasma levels of ammonia is also important and should be performed on a free-flowing venous or arterial blood sample collected into a lithium heparin or EDTA tube and transported on ice to the laboratory. The sample must be processed within 15 min of draw and analysed immediately.

Once hyperammonaemia is identified, protein intake must be temporarily stopped and plasma ammonia levels monitored every 3 h (ref.^10^). In a patient with an ammonia level at the upper limit of normal for their age — that is, 110 μmol/l (154 μg/dl) at age 1–7 days, <90 μmol/l (126 μg/dl) at age 8–14 days and 16–53 µmol/l (22–74 μg/dl) at age 15 days to adult — stopping protein intake and initiating intravenous glucose and lipids to prevent catabolism is generally adequate. Crucially, protein intake must be reintroduced within a maximum of 48 h following the return of ammonia levels to 80–100 μmol/l (136–170 μg/dl) in order to avoid catabolism^23^. Two factors determine the prognosis of neurological damage: the duration of hyperammonaemic coma and plasma ammonia levels^25,26^. Adverse prognostic factors include hyperammonaemic coma lasting >3 days, increased intracranial pressure and/or a plasma ammonia level >1,000 μmol/l (1,703 μg/dl)^15^. In a study of 22 children with UCDs who survived a hyperammonaemic coma, the duration of coma was inversely correlated with the children’s IQ at 12 months after recovery from the hyperammonaemic coma^27,28^. The severity of abnormalities on brain CT also correlated with the duration of coma^29^. Early management and reduced duration of hyperammonaemic coma may prevent adverse neurological outcomes^29^. The initial medical and nutritional management of hyperammonaemia is summarized in Box 1.

Box 1 Medical management of hyperammonaemia^30^ Stop protein intake.

- i.v. glucose: infusion rate 8–10 mg/kg/min
- i.v. lipids: 0.5 g/kg daily, up to 3 g/kg daily
- Caloric intake: ≥100 kcal/kg daily
- i.v. sodium benzoate: maximum 12 g daily (high-dose benzoate can be toxic and lethal within 1 h) given over 90 min as bolus then as maintenance over 24 h:
  - weight <20 kg, 250 mg/kg
  - weight >20 kg, 5.5 g/m^2^
- i.v. sodium phenylacetate, given over 90 min as bolus then as maintenance over 24 h:
  - weight <20 kg, 250 mg/kg
  - weight >20 kg, 5.5 g/m^2^
- i.v. sodium benzoate and sodium phenylacetate, given over 90–120 min as bolus then as maintenance over 24 h:
  - weight <20 kg, 250 mg/kg
  - weight >20 kg, 5.5 g/m^2^
- i.v. l-arginine hydrochloride, given over 90 min as bolus then as maintenance over 24 h:
  - weight <20 kg; 200 mg/kg in patients with OTC and CPS deficiencies, 600 mg/kg in patients with ASS and ASL deficiencies
  - weight >20 kg; 4 g/m^2^ in patients with OTC and CPS deficiencies, 12 g/m^2^ in patients with ASS and ASL deficiencies
- i.v. l-carnitine: 50 mg/kg loading dose given over 90 min, then 100–300 mg/kg daily (not needed in patients with UCDs but needed in patients with organic acidaemias)
- Vitamins (B_12_ 1 mg, biotin 5–15 mg)
- Oral phenylbutyrate (after UCD diagnosis)

ASL, argininosuccinate lyase; ASS, argininosuccinate synthase; CPS, carbamoyl phosphate synthase I; i.v., intravenous; OTC, ornithine transcarbamylase; UCD, urea cycle disorder.

### NKRT guidelines

NKRT is generally indicated at serum ammonia levels >150 μmol/l (255 μg/dl). The goal of NKRT is to provide an alternative route for nitrogen excretion (that is, its sequestration by nitrogen-scavenging agents)^2^. Nitrogen scavengers include sodium benzoate, sodium phenylacetate, sodium phenylbutyrate (a precursor of phenylacetate) and glycerol phenylbutyrate. Benzoate conjugates with glycine to generate hippurate, whereas phenylacetate conjugates with glutamine to generate phenylacetylglutamine^23,30^. Intravenous sodium benzoate and sodium phenylacetate can often be administered much more rapidly than KRT. These agents can also be administered in conjunction with KRT but will be dialysed along with other small molecules. Nonetheless, in one infant with hyperammonaemia due to a suspected inborn error of metabolism, the use of a cocktail of intravenous nitrogen scavengers followed by sequential HD and haemofiltration successfully corrected the hyperammonaemia despite rapid clearance of the nitrogen scavengers by dialysis^31^.

Additionally, urea cycle intermediates such as l-arginine or l-citrulline are primers of the urea cycle that can be supplemented to aid in ammonia removal. The choice of intermediate depends on which deficiency is present. l-Arginine is also a precursor to nitric oxide, a potent vasodilator; intravenous administration of arginine could therefore lead to hypotension. The arginine dose might need to be titrated or reduced, especially if the patient is simultaneously receiving HD. In patients with ammonia levels >150 μmol/l (255 μg/dl), the protocol depends on the blood ammonia level and whether or not the patient has a known UCD. Treatment of hyperammonaemia based on the ammonia level in both undiagnosed patients and patients with known UCDs should be initiated according to the guidelines for NKRT management^23,30^. Box 2 presents the consensus panel’s recommendations for NKRT.

Box 2 Recommendations from the consensus panel for NKRT management

- Consensus recommendation 1: immediately conduct further investigations, without delaying treatment, when elevated ammonia levels are detected (evidence level 4D)
- Consensus recommendation 2: discontinue all oral feeds and provide adequate calories (≥100 kcal/kg daily) as intravenous glucose and lipids (evidence level 4B)
- Consensus recommendation 3: maintain a glucose infusion rate of 8–10 mg/kg/min and provide lipids (0.5 g/kg daily, up to 3 g/kg daily) (evidence level 4B)
- Consensus recommendation 4: gradually reintroduce protein (by 0.25 g/kg daily, up to 1.5 g/kg daily) within 48 h (evidence level 4 C); if stabilization of serum ammonia levels takes longer than 48 h and protein is not supplied, protein catabolism will drive further ammonia production
- Consensus recommendation 5: use nitrogen-scavenging agents (such as sodium benzoate and sodium phenylacetate) and urea cycle intermediates (such as l-arginine and l-citrulline) at the recommended dosage (evidence level 4 C)
  - Intravenous sodium benzoate (maximum dose 12 g daily; high-dose benzoate can be toxic and lethal within 1 h): body weight <20 kg, 250 mg/kg; body weight >20 kg, 5.5 g/m^2^; given over 90 min as bolus then as maintenance over 24 h
  - Intravenous sodium phenylacetate: body weight <20 kg, 250 mg/kg; body weight >20 kg, 5.5 g/m^2^; given over 90 min as bolus then as maintenance over 24 h
  - Intravenous sodium benzoate and sodium phenylacetate: body weight <20 kg, 250 mg/kg; body weight >20 kg, 5.5 g/m^2^; given over 90–120 min as bolus then as maintenance over 24 h
  - Intravenous l-arginine hydrochloride: body weight <20 kg, 200 mg/kg for OTC and CPS deficiencies and 600 mg/kg for ASS and ASL deficiencies; weight >20 kg, 4 g/m^2^ for OTC and CPS deficiencies and 12 g/m^2^ for ASS and ASL deficiencies; given over 90 min as bolus then as maintenance over 24 h
  - Intravenous l-carnitine: 50 mg/kg loading dose given over 90 min, then 100–300 mg/kg daily (not needed for UCD but needed for organic aciduria)

ASL, argininosuccinate lyase; ASS, argininosuccinate synthase; CPS, carbamoyl phosphate synthase I; NKRT, non-kidney replacement therapy; OTC, ornithine transcarbamylase; UCD, urea cycle disorder.

### KRT guidelines

Ammonia is amenable to diffusive dialysis as it does not notably bind to albumin or other proteins and is a small molecule with a molecular weight of 17 Da^2^. Published indications for dialysis in neonates and children include serum ammonia level >500 μmol/l (852 μg/dl) or a lower level if there is an inadequate clinical response after 4 h of medical management^10,32^. If serum levels of ammonia are 100–300 μmol/l (170–511 μg/dl) and the patient shows clinical signs of severe encephalopathy and/or seizure, with consistent EEG findings, treatment with ammonia-scavenging agents (as recommended in Boxes 1 and 2) should be initiated. After 2 h, signs of seizures and ammonia levels should be reassessed; if the patient had an indeterminate response to ammonia-scavenging treatment dialysis should be considered. In patients with serum levels of ammonia 301–499 μmol/l (513–850 μg/dl) who show clinical signs of moderate to severe encephalopathy or seizure, scavenger treatment should be initiated while the patient is being prepared for dialysis. However, these numerical values should only serve as a general guide and should not be applied rigidly. Instead, the evolving clinical status of the patient should be the primary determinant of whether or not to begin KRT.

Institutional preference and local facilities determine the choice of dialysis modality. This decision is also influenced by the safety, efficacy and complications of each type of dialysis. Intermittent HD and CKRT have proved more efficacious than PD^15,23^. The decision as to whether to use HD, CKRT or PD should be made jointly by paediatric, internal medicine, nephrology, metabolism and critical care teams, as appropriate. The decision-making process should take into account the availability of dialysis equipment and/or staff, the diagnosis and overall condition of the patient, the trend in serum ammonia levels, the response to nitrogen-scavenger therapy and the age and body weight of the patient.

### Peritoneal dialysis guidelines

PD was the primary treatment for hyperammonaemia before the mid-1990s, when HD and CKRT were considered too risky and challenging to perform in neonates and children. However, data on the use of PD in this setting show that this technique has limited efficacy in the treatment of hyperammonaemia^33^, and the use of PD in the management of hyperammonaemia is now somewhat controversial.

A study comparing various modalities of KRT in eight patients with hyperammonaemia showed that PD was less efficient than intermittent HD or CKRT in reducing ammonia levels^34^. Improved outcomes were seen in patients with greater ultrafiltration flow rates; that is, in those treated with HD and CKRT rather than PD. In a 1976 case report of a neonate with argininosuccinic aciduria, PD was initiated on day 4 and resulted in an improvement in blood ammonia level from 432 μmol/l (736 μg/dl) to 176 μmol/l (300 μg/dl). However, after this initial fall in ammonia concentration, PD failed to lower the blood ammonia concentration any further^35^. In a retrospective analysis of data from six Italian centres describing children and neonates with hyperammonaemia treated with either PD or HD, intermittent HD removed ammonia more rapidly than PD, but no differences were observed in neurological sequelae or survival between patients who received PD and those who received HD^36^. In this study, clinical expertise and available resources dictated the ammonia level at which dialysis was initiated and what modality was chosen. The researchers recommended that PD should be performed in situations where extracorporeal therapies are not available or unsafe and that HD should be performed in patients who show a rapid rise in blood ammonia levels^36^. A PD protocol is provided in Supplementary Information 3. Box 3 lists recommendations from the consensus panel for PD.

Some centres, however, might lack the equipment to enable the use of other extracorporeal therapies and PD might remain the only treatment option. Although PD offers a quick alternative for the immediate management of hyperammonaemia if HD or CKRT are not available^37^, complications of PD include obstruction and leakage of the catheters, which can result in delayed toxin clearance and increased duration of dialysis^38^. Additionally, few devices for PD are suitable for the treatment of hyperammonaemia in small children. These devices are not always promptly commercially available, and their availability mainly depends on the local economic context and established practices. In turn, device availability is a factor that contributes to determining the expertise of local operators.

Box 3 Recommendations from the consensus panel for PD

- Consensus recommendation 6: PD is recommended when other modalities of KRT are unavailable (evidence level 3B).
- Consensus recommendation 7: rigid peritoneal catheters are not recommended as they are associated with increased rates of complications such as clotting and infections (evidence level 3B).
- Consensus recommendation 8: if PD is the only available modality, the consensus panel recommends its use in the following indications (evidence level 3C):
  - Rapidly deteriorating neurological status, coma, or cerebral oedema
  - Persistently high blood ammonia levels >400 μmol/l (681 μg/dl) refractory to NKRT medical measures
  - Rapid rise in ammonia levels >300 μmol/l (511 μg/dl) within a few hours that cannot be controlled via NKRT medical measures

KRT, kidney replacement therapy; NKRT, non-kidney replacement therapy; PD, peritoneal dialysis.

### CKRT guidelines

The introduction of CKRT (which includes CVVH and CVVHDF) led to improvements in the outcomes of patients with hyperammonaemia. Compared with HD, CKRT results in fewer cardiovascular complications, less need for plasma and blood transfusions, and a lower risk of rebound hyperammonaemia^34,36,39^. Owing to its continuous nature, CKRT is not associated with major fluid or osmotic shifts and therefore has a reduced likelihood of aggravating the raised intracranial pressure associated with hyperammonaemia^40^. The need to obtain vascular access is the main limitation to the use of CKRT.

Continuous venovenous HD (CVVHD) is superior to conventional HD and PD in infants owing to its ability to maintain haemodynamic stability by removing isotonic fluid^40^. All forms of CKRT are safe and efficacious methods for the management of hyperammonaemia; however, CVVHD enables a higher ammonia clearance rate than CVVH. A case report described two severely symptomatic neonates, aged 5 days and 6 days, with blood ammonia levels of 881 μmol/l (1,500 μg/dl) and 776 μmol/l (1,322 μg/dl), respectively, who were later diagnosed with OTC deficiency^41^. Both patients were admitted to the neonatal intensive care unit. The 5-day-old patient presented with cyanosis and gasping and was intubated and started on CKRT, given his haemodynamic instability. The ammonia level of the patient decreased to 367  μmol/l (625 μg/dl) within 2 h and he exhibited spontaneous movement and neurological improvement within 1 h of starting CKRT^41^. The 6-day-old patient presented with poor feeding, vomiting and somnolence, and was treated with high-dose CKRT in view of a pretreatment blood ammonia level of 1,387 μmol/l (2,362 μg/dl). The patient’s ammonia levels decreased dramatically to 90 μmol/l (153 μg/dl) within 7 h of treatment, rebounded to 402 μmol/l (685 μg/dl) within 10 h, but remained <100 μmol/l (170 μg/dl) over the next 24 h without further KRT. After 6 months, this patient underwent liver transplantation^41^. These case reports show that CVVHD results in greater ammonia clearance than CVVH. In neonates undergoing CKRT, the use of a warmed dialysate provides added haemodynamic stability^42^. Although fewer complications are associated with CKRT than with other forms of dialysis, the pre-CKRT status of each individual is a major determinant of survival. A retrospective study showed that the most important prognostic factor was the duration of hyperammonaemic coma prior to the start of dialysis^27^; by contrast, patient outcomes were not influenced by the rate of ammonia clearance^36^.

In patients with blood ammonia levels >1,500 μmol/l (2,555 μg/dl), high-dose CKRT (maximal blood flow rate (Qb) 30–50 ml/min; dialysate flow rate (Qd)/Qb >1.5) can be initiated, which enables rapid clearance of ammonia and reduces the need to switch between HD and CKRT modalities. Experimental and clinical evidence indicates that a Qd >1,000 ml/h is required to exploit the maximum potential of CKRT in neonates^38^. Although rebound hyperammonaemia has also been reported with high-dose CKRT, this complication occurs to a lesser extent than in HD and does not require further KRT management. Furthermore, CKRT can be completed in a single dialysis run without the need for a change in equipment. In two patients with OTC deficiency, rapid removal of ammonia was seen after initiation of high-dose CKRT — Qb 10–20 ml/min in neonates and infants or, in older children, the maximum blood flow allowed by the catheter. This high-dose CKRT resulted in clearance rates (that is, total replacement and/or Qd) of up to 8,000 ml/1.73 m^2^/h or more and was safe for the patients^34^. In other studies, Qb 10–20 ml/min in neonates and infants has achieved clearance rates (total replacement and/or Qd) of ≥2,500 ml/1.73 m^2^/h^41,43^. However, not all CKRT machines can achieve an optimal Qd value. One CKRT device (Asahi Sigma Plasauto) allows a Qd of up to 6 l/h, whereas the Prismaflex and Fresenius devices (paediatric circuits) limit Qd to 1 l/h. Overall, these Qd restrictions limit the efficiency of CKRT and create the need for additional HD in patients with very high ammonia levels. Additionally, the literature shows that problems with vascular access, heparinization, electrolyte alterations and clotting of the membrane and/or circuit may lead to inadequate CKRT (that is, delivered other than as prescribed in the recommendations below). Protocols for CKRT and high-dose CKRT are included in Supplementary Information 3. Recommendations from the consensus panel for CKRT are presented in Box 4.

Box 4 Recommendations from the consensus panel for CKRT

- Consensus recommendation 9: CKRT, specifically high-dose CVVHD, is the recommended first-line treatment for hyperammonaemia when possible (evidence level 3B).
- Consensus recommendation 10: CKRT should be initiated in a patient with hyperammonaemia in the following situations:
  - Rapidly deteriorating neurological status, coma, or cerebral oedema with blood ammonia level >150 μmol/l (256 μg/dl) (evidence level 3B)
  - Presence of either moderate or severe encephalopathy (evidence level 3B). Moderate encephalopathy is defined as lethargy, distal flexion, decreased activity, complete extension, hypotonia, weak suck or incomplete Moro reflex, constricted pupils or bradycardia^69^. Severe encephalopathy is defined as stupor or coma, no activity, decerebrate posture, flaccid tone, absent suck, absent Moro reflex, pupils non-reactive to light, variable heart rate or sleep apnoea^69^.
  - Persistently high blood ammonia levels >400 μmol/l (681 μg/dl) refractory to NKRT medical measures (evidence level 3B)
  - Rapid rise in blood ammonia levels to >300 μmol/l (511 μg/dl) within a few hours that cannot be controlled via NKRT medical therapies (evidence level 3B)
- Consensus recommendation 11: warming the dialysate helps maintain haemodynamic stability in patients who receive CKRT (evidence level 3B).
- Consensus recommendation 12: high-dose CKRT with Qb 30–50 ml/min, aiming at Qd/Qb >1.5, may be used for the initial treatment of patients with a blood ammonia level >1,000 μmol/l (1,703 μg/dl) (evidence level 4D).
- Consensus recommendation 13: step-down CKRT can follow HD or high-dose CKRT when the blood ammonia level is <200 μmol/l (341 μg/dl) on at least two once-hourly measurements, keeping in mind that therapy with nitrogen-scavenging agents might be sufficient to prevent rebound hyperammonaemia (evidence level 4D).

CKRT, continuous kidney replacement therapy; CVVHD, continuous venovenous haemodialysis; HD, haemodialysis; NKRT, non-kidney replacement therapy; Qb, blood flow rate; Qd, dialysis fluid flow rate.

### Haemodialysis guidelines

Intermittent HD can decrease blood ammonia concentrations by 75% within 3–4 h (ref.^37^). The main limitations of this method in neonates are the risk of rebound hyperammonaemia and the need to obtain vascular access, although such access has been achieved with the use of a peripheral artery and umbilical vein^44^. A 2007 study comparing different forms of KRT in eight infants with hyperammonaemia found that patients receiving intermittent HD showed a 50% reduction in ammonia levels after 1–2 h, whereas patients treated with CVVHD took 2–14 h to show this level of reduction^34^. However, the presence of rebound hyperammonaemia often necessitates treatment with multiple HD sessions, which can cause hypotension and rapid osmotic shifts that further compromise the haemodynamic stability of the patient^44^. The hypotension associated with the use of intermittent HD can also worsen cerebral oedema and increase the chance of cerebral herniation in patients with raised intracranial pressure^45^. However, in low-body-weight infants, short-duration, intermittent HD is a safe and effective way to ensure rapid reversal of hyperammonaemia and enable the patient to recover between dialysis sessions, which prevents HD-related prolonged hypotension, membrane clotting and prolonged heparinization^46^. The rapid shifts in osmolarity associated with HD also increase the risk of further elevations in intracranial pressure, especially in neonates. Thus, in these patients, osmolarity should be monitored regularly during HD, and the dialysate formula should be modified to minimize or avoid any rapid shifts in osmolarity^47^.

In patients with inborn errors of metabolism, high HD infusion rates enable the administration of calories (along with ammonia clearance) to prevent catabolism^41,48^. An HD protocol is listed in Supplementary Information 3. Box 5 presents recommendations from the consensus panel for HD.

Box 5 Recommendations from the consensus panel for HD

- Consensus recommendation 14: intermittent HD is recommended in patients who require rapid ammonia clearance (evidence level 3B).
- Consensus recommendation 15: for hyperammonaemia, initiation of intermittent HD is recommended in the following situations:
  - Rapidly deteriorating neurological status, coma, or cerebral oedema (evidence level 3B)
  - HD or high-dose CKRT may be used as initial therapy in patients with blood ammonia levels >1,000 μmol/l (1,703 μg/dl) (evidence level 4D).

CKRT, continuous kidney replacement therapy; HD, haemodialysis.

### Hybrid therapy guidelines

The use of HD or CKRT alone is effective in treating and reducing toxic ammonia levels. However, combinations of HD and CKRT, known as hybrid or sequential therapy, can gradually reduce ammonia levels while controlling the rebound effect. The results of a retrospective study showed that HD should be the first-line KRT modality for rapidly reducing ammonia levels, followed by CKRT to prevent any rebound^49^. Another advantage of CKRT is the ability to safely replace electrolytes lost during dialysis, which is not possible in intermittent HD. Accordingly, hybrid therapy is initiated with HD when ammonia levels are >1,500 μmol/l (2,555 μg/dl), then transitioned to CKRT^1,41^. Step-down CKRT can follow either HD or high-dose CKRT once ammonia levels are <200 μmol/l (280 μg/dl) on at least two consecutive hourly measurements. Nitrogen-scavenging agents should be instituted once the patient is off HD or high-dose CKRT to prevent rebound.

Another hybrid method of KRT is CKRT with ECMO support. In a neonate requiring urgent dialysis, the need for a small catheter size and the small volume of the dialysate circuit can limit ammonia clearance. ECMO is primarily used in patients with cardiorespiratory failure, although a few studies have shown the need for ECMO in low-birth-weight neonates with hyperammonaemia and severe hypotension requiring HD^50–52^. The use of CKRT with ECMO can increase the blood volume of the patient, enables the use of a larger cannula, avoids haemodynamic instability and leads to the rapid clearance of ammonia. ECMO-assisted CVVHD results in improved haemodynamic stability and safer dialysis in neonates, but carries an increased risk of causing a substantial cerebrovascular event. This risk is particularly elevated in low-birth-weight neonates, possibly owing to poor heart function, increased cerebral blood flow and increased intracranial pressure^53,54^.

An alternative form of hybrid therapy consists of therapeutic hypothermia combined with various KRT modalities. This approach is based on the recognition that whole-body therapeutic hypothermia can slow down ammonia production by decreasing metabolism throughout the body. Each 1 °C decrease in body temperature reduces the basal metabolic rate by 8%^54^. A pilot study testing various approaches to KRT combined with therapeutic hypothermia showed a faster decline and earlier stabilization of ammonia levels when the same KRT modality was compared in therapeutically cooled versus normothermic patients^55^. Further studies are required to determine the true effects of therapeutic cooling on the reduction of ammonia levels and the improvement in neurological outcomes in patients with hyperammonaemia. Box 6 presents recommendations from the consensus panel for hybrid therapy.

Box 6 Recommendations from the consensus panel for hybrid therapy

- Consensus recommendation 16: HD or CKRT combined with ECMO is recommended in neonates, especially those who are haemodynamically unstable. The combination of HD or CKRT with ECMO increases the patient’s blood volume, enables the use of a larger cannula and facilitates improved haemodynamic control (evidence level 4D).
- Consensus recommendation 17: HD or CKRT combined with ECMO is suggested for the treatment of hyperammonaemia in the following situations:
  - Haemodynamic instability in a small neonate with poor vascular access for standard CKRT (evidence level 4D)
  - Rapidly deteriorating neurological status, coma, or cerebral oedema (evidence level 4D)
  - Presence of moderate or severe encephalopathy. Moderate encephalopathy is defined as lethargy, distal flexion, decreased activity, complete extension, hypotonia, weak suck or incomplete Moro reflex, constricted pupils, or bradycardia^69^. Severe encephalopathy is defined as stupor or coma, no activity, decerebrate posture, flaccid tone, absent suck, absent Moro reflex, pupils non-reactive to light, variable heart rate or sleep apnoea^69^ (evidence level 4D).
  - Persistently high blood ammonia levels >400 μmol/l (681 μg/dl) refractory to NKRT medical measures (evidence level 4D)
  - A rapid rise in ammonia levels to >300 μmol/l (511 μg/dl) within a few hours that cannot be controlled via NKRT medical therapies (evidence level 4D)

CKRT, continuous kidney replacement therapy; ECMO, extracorporeal membrane oxygenation; HD, haemodialysis; NKRT, non-kidney replacement therapy.

## Conclusions

A high index of suspicion for hyperammonaemia is mandatory in paediatric patients of any age with suggestive clinical symptoms, given that almost all survivors have developmental disabilities that correlate with the number, severity and duration of hyperammonaemic episodes. Prompt treatment with KRT and/or NKRT, the choice of which depends on the ammonia concentrations and presenting symptoms of the patient, is crucial. The paediatric literature with regard to hyperammonaemia requiring KRT is limited. To our knowledge, a consensus recommendation including a literature review with expert guideline recommendations has not yet been published. More studies are needed to strengthen the recommendations presented in this article; these recommendations will be followed by an audit.

## Supplementary information

Supplementary information

## References

[1] Auron A, Brophy PD (2012). Hyperammonemia in review: pathophysiology, diagnosis, and treatment. Pediatr. Nephrol..

[2] Gupta S, Fenves AZ, Hootkins R (2016). The role of RRT in hyperammonemic patients. Clin. J. Am. Soc. Nephrol..

[3] Upadhyay R, Bleck TP, Busl KM (2016). Hyperammonemia: what urea-lly need to know: case report of severe noncirrhotic hyperammonemic encephalopathy and review of the literature. Case Rep. Med..

[4] Mew, N. A., Pappa, M. B., Gropman, A. L. in *Rosenberg’s Molecular and Genetic Basis of Neurological and Psychiatric Disease* (eds Rosenberg, R. N. & Pascual, J. M.) 633–647 (Elsevier, 2014).

[5] Najafi R (2016). Demographic and clinical findings in pediatric patients affected by organic acidemia. Iran. J. Child. Neurol..

[6] American College of Medical Genetics Newborn Screening Expert Group (2006). Newborn screening: toward a uniform screening panel and system–executive summary. Pediatrics.

[7] Coude FX, Sweetman L, Nyhan WL (1979). Inhibition by propionyl-coenzyme A of *N*-acetylglutamate synthetase in rat liver mitochondria. A possible explanation for hyperammonemia in propionic and methylmalonic acidemia. J. Clin. Invest..

[8] Dercksen M (2014). Inhibition of N-acetylglutamate synthase by various monocarboxylic and dicarboxylic short-chain coenzyme A esters and the production of alternative glutamate esters. Biochim. Biophys. Acta.

[9] Dionisi-Vici C (2002). Inborn errors of metabolism in the Italian pediatric population: a national retrospective survey. J. Pediatr..

[10] Alfadhel M (2016). Guidelines for acute management of hyperammonemia in the Middle East region. Ther. Clin. Risk Manag..

[11] Rangroo Thrane V (2013). Ammonia triggers neuronal disinhibition and seizures by impairing astrocyte potassium buffering. Nat. Med..

[12] Albrecht J, Norenberg MD (2006). Glutamine: a Trojan horse in ammonia neurotoxicity. Hepatology.

[13] Nagamani SC (2012). Nitric-oxide supplementation for treatment of long-term complications in argininosuccinic aciduria. Am. J. Hum. Genet..

[14] Gardeitchik T, Humphrey M, Nation J, Boneh A (2012). Early clinical manifestations and eating patterns in patients with urea cycle disorders. J. Pediatr..

[15] Burton BK (1998). Inborn errors of metabolism in infancy: a guide to diagnosis. Pediatrics.

[16] Tuchman M (2008). Cross-sectional multicenter study of patients with urea cycle disorders in the United States. Mol. Genet. Metab..

[17] Lavergne V (2012). The EXTRIP (extracorporeal treatments in poisoning) workgroup: guideline methodology. Clin. Toxicol..

[18] Fitch, K. et al. The RAND/UCLA appropriateness method user’s manual. *RAND Corporation* https://www.rand.org/content/dam/rand/pubs/monograph_reports/2011/MR1269.pdf (2001).

[19] Thornton J (2013). Introducing GRADE across the NICE clinical guideline program. J. Clin. Epidemiol..

[20] Moher D, Liberati A, Tetzlaff J, Altman DG, The PRISMA Group (2009). Preferred reporting items for systematic reviews and meta-analyses: the PRISMA statement. PLoS Med..

[21] Higgins JP (2011). The Cochrane Collaboration’s tool for assessing risk of bias in randomised trials. BMJ.

[22] Wells, G. P. et al. The Newcastle-Ottawa Scale (NOS) for assessing the quality of nonrandomised studies in meta-analyses. *The Ottawa Hospital* http://www.ohri.ca/programs/clinical_epidemiology/oxford.asp (2013).

[23] Haberle J (2012). Suggested guidelines for the diagnosis and management of urea cycle disorders. Orphanet J. Rare Dis..

[24] Haberle J (2011). Clinical practice: the management of hyperammonemia. Eur. J. Pediatr..

[25] Haberle J (2013). Clinical and biochemical aspects of primary and secondary hyperammonemic disorders. Arch. Biochem. Biophys..

[26] Colombo JP, Peheim E, Kretschmer R, Dauwalder H, Sidiropoulos D (1984). Plasma ammonia concentrations in newborns and children. Clin. Chim. Acta.

[27] Batshaw ML (1984). Hyperammonemia. Curr. Probl. Pediatr..

[28] Batshaw ML, Tuchman M, Summar M, Seminara J, Members of the Urea Cycle Disorders Consortium (2014). A longitudinal study of urea cycle disorders. Mol. Genet. Metab..

[29] Msall M, Batshaw ML, Suss R, Brusilow SW, Mellits ED (1984). Neurologic outcome in children with inborn errors of urea synthesis. Outcome of urea-cycle enzymopathies. N. Engl. J. Med..

[30] Batshaw ML, MacArthur RB, Tuchman M (2001). Alternative pathway therapy for urea cycle disorders: twenty years later. J. Pediatr..

[31] Bunchman TE (2007). Phenylacetate and benzoate clearance in a hyperammonemic infant on sequential hemodialysis and hemofiltration. Pediatr. Nephrol..

[32] Picca S, Bartuli A, Dionisi-Vici C (2008). Medical management and dialysis therapy for the infant with an inborn error of metabolism. Semin. Nephrol..

[33] Jouvet, P. & Schaefer, F. in *Pediatric Dialysis* Vol. **2** (eds Warady, B. A., Schaefer, F. & Alexander, S. R.) 765–774 (Springer, 2012).

[34] Lai YC, Huang HP, Tsai IJ, Tsau YK (2007). High-volume continuous venovenous hemofiltration as an effective therapy for acute management of inborn errors of metabolism in young children. Blood Purif..

[35] Francois B, Cornu G, de Meyer R (1976). Peritoneal dialysis and exchange transfusion in a neonate with argininosuccinic aciduria. Arch. Dis. Child..

[36] Picca S (2015). Short-term survival of hyperammonemic neonates treated with dialysis. Pediatr. Nephrol..

[37] Bilgin L, Unal S, Gunduz M, Uncu N, Tiryaki T (2014). Utility of peritoneal dialysis in neonates affected by inborn errors of metabolism. J. Paediatr. Child. Health.

[38] Schaefer F, Straube E, Oh J, Mehls O, Mayatepek E (1999). Dialysis in neonates with inborn errors of metabolism. Nephrol. Dial. Transpl..

[39] Hiroma T, Nakamura T, Tamura M, Kaneko T, Komiyama A (2002). Continuous venovenous hemodiafiltration in neonatal onset hyperammonemia. Am. J. Perinatol..

[40] Osgood M, Muehlschlegel S (2017). Point: should continuous venovenous hemofiltration always be the preferred mode of renal replacement therapy for the patient with acute brain injury? Yes. Chest.

[41] Spinale JM, Laskin BL, Sondheimer N, Swartz SJ, Goldstein SL (2013). High-dose continuous renal replacement therapy for neonatal hyperammonemia. Pediatr. Nephrol..

[42] Chan WK, But WM, Law CW (2002). Ammonia detoxification by continuous venovenous haemofiltration in an infant with urea cycle defect. Hong Kong Med. J..

[43] Hanudel M, Avasare S, Tsai E, Yadin O, Zaritsky J (2014). A biphasic dialytic strategy for the treatment of neonatal hyperammonemia. Pediatr. Nephrol..

[44] Kaneko M (2013). Continuous hemodialysis therapy for an extremely low-birthweight infant with hyperammonemia. Pediatr. Int..

[45] Davenport A, Will EJ, Davison AM (1990). Early changes in intracranial pressure during haemofiltration treatment in patients with grade 4 hepatic encephalopathy and acute oliguric renal failure. Nephrol. Dial. Transpl..

[46] Rajpoot DK, Gargus JJ (2004). Acute hemodialysis for hyperammonemia in small neonates. Pediatr. Nephrol..

[47] Liotta EM (2018). Osmotic shifts, cerebral edema, and neurologic deterioration in severe hepatic encephalopathy. Crit. Care Med..

[48] Haller M, Henzler-Le Boulanger A, Sass JO, Brandis M, Zimmerhackl LB (2005). Successful extracorporeal treatment of a male with hyperammonaemic coma. Nephrol. Dial. Transpl..

[49] McBryde KD (2006). Renal replacement therapy in the treatment of confirmed or suspected inborn errors of metabolism. J. Pediatr..

[50] Wen JX (2016). Continuous venovenous hemodialysis via extracorporeal membrane oxygenation pump for treatment of hyperammonemia secondary to propionic acidemia in monochorionic diamniotic twin boys. J. Pediatr..

[51] Summar M, Pietsch J, Deshpande J, Schulman G (1996). Effective hemodialysis and hemofiltration driven by an extracorporeal membrane oxygenation pump in infants with hyperammonemia. J. Pediatr..

[52] Robinson JR (2018). Rapid resolution of hyperammonemia in neonates using extracorporeal membrane oxygenation as a platform to drive hemodialysis. J. Perinatol..

[53] Gander JW, Rhone ET, Wilson WG, Barcia JP, Sacco MJ (2017). Veno-venous extracorporeal membrane oxygenation for continuous renal replacement in a neonate with propionic acidemia. J. Extra Corpor. Technol..

[54] Polderman KH, Herold I (2009). Therapeutic hypothermia and controlled normothermia in the intensive care unit: practical considerations, side effects, and cooling methods. Crit. Care Med..

[55] Lichter-Konecki U (2013). Feasibility of adjunct therapeutic hypothermia treatment for hyperammonemia and encephalopathy due to urea cycle disorders and organic acidemias. Mol. Genet. Metab..

[56] Pela I (2008). Peritoneal dialysis in neonates with inborn errors of metabolism: is it really out of date?. Pediatr. Nephrol..

[57] Vats A, Kashtan CE, Tuchman M, Mauer M (1998). Hemodialysis catheter placement and recirculation in treatment of hyperammonemia. Pediatr. Nephrol..

[58] Aygun F (2018). The impact of continuous renal replacement therapy for metabolic disorders in infants. Pediatr. Neonatol..

[59] Arbeiter AK (2010). Continuous venovenous haemodialysis (CVVHD) and continuous peritoneal dialysis (CPD) in the acute management of 21 children with inborn errors of metabolism. Nephrol. Dial. Transpl..

[60] Chen CY, Tsai TC, Lee WJ, Chen HC (2007). Continuous hemodiafiltration in the treatment of hyperammonemia due to methylmalonic acidemia. Ren. Fail..

[61] Ponikvar R (2002). Continuous renal replacement therapy and plasma exchange in newborns and infants. Artif. Organs.

[62] Kim HJ (2011). Acute treatment of hyperammonaemia by continuous renal replacement therapy in a newborn patient with ornithine transcarbamylase deficiency. Korean J. Pediatr..

[63] Westrope C, Morris K, Burford D, Morrison G (2010). Continuous hemofiltration in the control of neonatal hyperammonemia: a 10-year experience. Pediatr. Nephrol..

[64] Wong KY, Wong SN, Lam SY, Tam S, Tsoi NS (1998). Ammonia clearance by peritoneal dialysis and continuous arteriovenous hemodiafiltration. Pediatr. Nephrol..

[65] Braun MC, Welch TR (1998). Continuous venovenous hemodiafiltration in the treatment of acute hyperammonemia. Am. J. Nephrol..

[66] Enkai S (2003). Experience of continuous haemodiafiltration in a male neonate with ornithine transcarbamylase deficiency. Eur. J. Pediatr..

[67] Kosho T, Nakamura T, Kaneko T, Tamura M (2008). A case of neonatal-onset carbamoyl-phosphate synthase I deficiency treated by continuous haemodiafiltration. Eur. J. Pediatr..

[68] Lazier J, Lupichuk SM, Sosova I, Khan AA (2014). Hyperammonemic encephalopathy in an adenocarcinoma patient managed with carglumic acid. Curr. Oncol..

[69] Shankaran S (2002). Whole-body hypothermia for neonatal encephalopathy: animal observations as a basis for a randomized, controlled pilot study in term infants. Pediatrics.

