# Supplementary material for: Consensus guidelines for management of hyperammonaemia in paediatric patients receiving continuous kidney replacement therapy
Source: Nat Rev Nephrol. 2020 Apr 8;16(8):471–82. doi: 10.1038/s41581-020-0267-8 (PMC7366888; doi:10.1038/s41581-020-0267-8)
Supplement: Supplementary file 1 — Supplementary information [file 41581_2020_267_MOESM1_ESM.pdf]

nature REVIEWS NEPHROLOGY

SUPPLEMENTARY INFORMATION

In format as provided by the authors

Consensus guidelines for management of hyperammonaemia in paediatric patients receiving continuous kidney replacement therapy

Rupesh Raina, Jirair K. Bedoyan, Uta Lichter-Konecki, Philippe Jouvet, Stefano Picca, Nicholas Ah Mew, Marcel C. Machado, Ronith Chakraborty, Meghana Vemuganti, Manpreet K. Grewal, Timothy Bunchman, Sidharth Kumar Sethi, Vinod Krishnappa, Mignon McCulloch, Khalid Alhasan, Arvind Bagga, Rajit K. Basu, Franz Schaefer, Guido Filler and Bradley A. Warady

https://doi.org/10.1038/s41581-020-0267-8

Supplementary Information

Supplementary Box 1: Search Strategy

Ovid MEDLINE = 173
Ovid EMBASE = 299
Cochrane Central = 5
Total = 477
Duplicates removed = 148
Total to review = 329

Database: Ovid MEDLINE Epub Ahead of Print, In-Process & Other Non-Indexed Citations, Ovid MEDLINE Daily, Ovid MEDLINE and Versions <1946 to February 21, 2018> Search Strategy:

1 renal replacement therapy/ or exp renal dialysis/
2 ((kidney or renal) adj3 replacement adj3 therap*).mp.
3 (hemofiltrat* or haemodialys* or dialysis or "peritoneal dialysis" or capd or hemodiafiltrat*or haemoperfus*).mp.
4 1 or 2 or 3
5 Hyperammonemia/
6 hyperammon?emi*.mp.
7 4 and (5 or 6)
8 limit 7 to "all child (0 to 18 years)"
9 7 and (neonat* or newborn* or infan* or child* or adolescen*).mp,jw.
10 7 and (pediatr* or paediatr*).mp,jw.
11 8 or 9 or 10

Database: EMBASE <1974 to 2018 February 27> Search Strategy:

1 exp renal replacement therapy/
2 ((kidney or renal) adj3 replacement adj3 therap*).mp.
3 (h?emofiltrat* or h?emodialys* or dialysis or "peritoneal dialysis" or capd or h?emodiafiltrat*or h?emoperfus*).mp.
4 1 or 2 or 3
5 hyperammonemia/
6 hyperammon?emi*.mp.
7 4 and (5 or 6)
8 limit 7 to (embryo <first trimester> or infant <to one year> or child <unspecified age> or preschool child <1 to 6 years> or school child <7 to 12 years> or adolescent <13 to 17 years>)
9 7 and (neonat* or newborn* or infan* or child* or adolescen*).mp,jw.
10 7 and (pediatr* or paediatr*).mp,jw.
11 8 or 9 or 10
12 limit 11 to embase

Cochrane Central through Wiley Online Library

#1 [mh ^"renal replacement therapy"] or [mh "renal dialysis"]
#2 (kidney or renal) near/3 replacement near/3 therap*

#3 hemofiltrat* or haemofiltrat* or hemodialys* or haemodialys* or dialysis or "peritoneal dialysis" or capd or hemodiafiltrat* or haemodiafiltrat* or hemoperfus* or haemoperfus*
#4 #1 or #2 or #3
#5 [mh hyperammonemia]
#6 hyperammonemi* or hyperammonaemi*
#7 #4 and (#5 or #6)
#8 #7 and (neonat* or newborn* or infan* or child* or adolescen* or pediatr* or paediatr*) in Trials

Supplementary Table 1: PRISMA checklist[20]

Section or topic	Checklist item response
Background	An explicit statement of questions being addressed with reference to participants, interventions, comparisons, outcomes, and study designs (PICOS)
Protocol and registration	Indicates if a review protocol exists and if and when it can be accessed
	Indicates if the protocol has been registered, and if so, provides registration number
Eligibility criteria	Specifies study characteristics (e.g., PICOS, length of follow-up) and reports characteristics (e.g. years considered, language, publication status used as criteria for eligibility
Information sources	Describes all information sources (e.g. database with dates of coverage, contact with study authors to identify additional studies) in the search and the date last searched
Search	Presents full electronic search strategy for at least one database, including any limits used, such that it could be repeated
Study selection	States the process for selecting studies (e.g. screening, eligibility, included in systematic review and/or inclusion in meta-analyses)
Data collection process	Describes the method of data extraction from reports (e.g., piloted forms, independently, in duplicate) and any processes for obtaining and confirming data from investigators
Data items	Lists and defines all variables for which data were sought (e.g., PICOS, funding sources) and any assumptions and simplifications made
Risk of bias in individual studies	Describes methods used for assessing risk of bias of individual studies (including specification of whether this was done at the study or outcome level), and this information was used in any data synthesis
Summary measures	States the principal summary measures (e.g., risk ratios, difference in means)
Synthesis of results	Describes the methods of handling data and combining results of studies, if done, including measures of consistency (I^2) for each meta-analysis
Study selection	The numbers of studies screened, assessed for eligibility, and included in the review, with reasons for exclusion at each stage
Study characteristics	Characteristics for which data were extracted from each study (e.g., study size, PICOS, follow-up period)
Risk of bias across studies	Presents results of any assessment of risk of bias across studies
Additional analyses	Gives results of secondary analyses (e.g., sensitivity or sub-group analyses, meta-regression)

Supplementary Table 2: Expanded patient characteristics, interventions used and outcomes in included studies.

Study	(*n*), mean age	Indications (*n*)	KRT type, mean duration and ammonia levels after KRT	Ammonia clearance
**Bilgin et al. 2014, retrospective**	(9) 11.7 +/- 9.7 days	MSUD (3); Hyperammonemia peak range 531-1533μmol/L ((904.35–2,610.85 μg/dL): organic acidemias (4), UCD (2)	PD 4.6 ± 1.9 days; 300 ± 440 μmol/L (510.93 ± 749.36 μg/dL)	66%
**Pela et al. 2008, retrospective analysis**	(7) 2-3 days	CPS deficiency (1), PA (3), MMA (1), OTC deficiency (1), ASL (1); KRT initiated at hyperammonemia >1000 μmol/dL ((1,703.10 μg/dL)	PD (7) 2.4 days; range 5h - 15 days; <200mg/dL((340.62 μg/dL) after 20h KRT	57%
**Haller et al. 2005, case report**	(1) 2 days	Citrullinemia with peak hyperammonemia 874 μmol/L (1,488.51 μg/dL)	HD round 1: 4.5h; round 2: 2h; 588 μmol/L (1,001.42 μg/dL) after round 1; 89 μmol/L after round 2	100%
**Rajpoot & Gargus 2004, retrospective study**	(4) 7 h – 10 days	transient hyperammonemia (2), MMA (1), OTC deficiency (1); average hyperammonemia 595 μmol/L (1,013.34 μg/dL)	HD 4-5h; multiple sessions (2); 180 μmol/L (306.56 μg/dL)	100%
**Vats et al. 1998, case report**	(1) 2-year-old	CPS deficiency (1) with peak hyperammonemia 765 μmol/L (1,302.87 μg/dL)	HD 14h; 153 μmol/L (260.57 μg/dL)	100%
**Bunchman et al. 2007,** case r**eport**	(1) 3 days old	MMA with peak hyperammonemia 1,533 μmol/L (2,610.85 μg/dL)	HD and HF: 2h HD followed by 14h HF; 209 μmol/L (292.76 μg/dL)	100%
**Aygun et al. 2018, retrospective study**	(14) 5.5 +/- 7.4 months	OTC deficiency (5), MSUD (4), CPS deficiency (5)	CVVHDF (11) CVVHD (3): 16.6 +/- 15.6h; <200 μmol/L (340.62 μg/dL) (10)	85.70%
**Hanudel et al 2014, case report**	(2) 4 days	Citrullinemia with peak hyperammonemia to 841 umol/L (1,432.31 μg/dL). (1) MMA with peak hyperammonemia to 1,830 umol/L (116.67 μg/dL) (1).	CVVHD (2): 3 h high rate CKRT; 24h low rate CKRT; <100 μmol/L (170.31 μg/dL)	100%
**Spinale et al 2013, case report**	(2) 5 and 6 days	OTC deficiency (2) with peak hyperammonemia >1300μmol/L (2,214.03 μg/dL)	CVVHD (2): 7 h (1) 10 h. (1); <100 μmol/L (170.31 μg/dL); had mild rebound but then stayed below 100 μmol/L	100%
**Kim et al. 2011, case report**	(1) 3 days	OTC deficiency with peak hyperammonemia >1700mg/dL (998.18 μmol/L)	CVVHDF: 7 days; had two rounds due to rebound hyperammonemia; 200ug/dL(117.43 μmol/L)	0%

**Westrope et al**. **2010, retrospective study**	(14) 4 days	OTC deficiency (2); CPS deficiency (1); Citrullinemia (4); PA (5); MMA (2)	CVVH (13); CVVH and PD (1) 49 h (range 6-94 h); <200 µmol/L (340.62 µg/dL) (11)	64%
**Arbeiter et al**. **2010, retrospective analysis**	(21) 19 neonates 4.1 +/- 2.4 days; 1 year, 7 years	Citrullinemia (8), OTC deficiency (3), MMA (2), CPS deficiency (3), PA (1), Glutaric acidemia type II (1), Argininemia (1), unknown diagnosis (2); peak hyperammonemia 1225.8±1172.9 µmol/L (1717±1643 µg/dL)	CVVHD (17) 42 +/- 30.4 h; PD (4) 59.4 +/- 87.2 h; reduced by 50% within 4.7+/- 2.5 h with CVVHD; 13.5 +/- 6.2 h with PD	84% CVVHD; 50% PD
**Chen et al**. **2007, case report**	(3) 3-7 days	MMA (3); peak hyperammonemia 449 - 932 µg/dL	CAVHDF 19 h; 85–124 µg/dL	100%
**Ponikvar et al**. **2002, retrospective study**	(21) 15.7 +/- 11.7 days	Anuria with hyperhydration (17), azotemia with anuria (1), HUS (1 patient), and neonatal hyperammonemia (2) with peak ammonia >1300 µmol/L (2,214.03 µg/dL)	CVVH (12); CVVHD (1); CAVHF (1); CVVHDF (6): 66.8 +/- 56.6h; CVVHD: 347 µmol/L (590.98 µg/dL); CVVHDF: 104 µmol/L (177.12 µg/dL)	42.90%
**Hiroma et al**. **2002, case report**	(4) 2-3 days	CPS deficiency (1); MMA (1); pyruvate carboxylase deficiency (2) with hyperammonemia 260-1790 ug/dL (153.66-1,051.02 µmol/L)	CVVHDF (4) 30 h; < 200 µg/dL(117.43 µmol/L)	50%
**Chan et al**. **2002, case report**	(1) 15 days	CPS deficiency (1) with peak hyperammonemia 700 µmol/L (1,192.17 µg/dL)	CVVH 6 h; 105 µmol/L (178.83 µg/dL)	100%
**Schaefer et al**. **1999, retrospective analysis**	(12) 4 days–2 months	MSUD (4); PA (3); CPS deficiency (2); CPS deficiency (2); ASL deficiency (1) with peak hyperammonemia 3820 µmol/L (6,505.84 µg/dL)	CVVHD (7), PD (5)<br>CVVHD: mean 29.5 h; 50% ammonia reduction time 7.1h; PD: dwell time 30-60 min; mean 73 h; 50% ammonia reduction time 17.9h	83%
**Wong et al**. **1998, case report**	(1) 4 days	UCD (1) with peak hyperammonemia 1,970 µmol/L (3355.11 µg/dL)	CAVHD 7 cycles; 30 min cycles; <200 µmol/L (340.62 µg/dL)	0%
**Braun et al**. **1998, case report**	(1) 8 months	Cholestatic hepatitis with peak hyperammonemia 306 µmol/L (521.15 µg/dL)	CVVHD 48 hr; 86 µmol/L (146.47 µg/dL)	100%
**Enkai et al**. **2003, case report**	(1) 4 day	OTC deficiency with peak hyperammonemia 1903 µmol/L (3241 µg/dL)	CHDF Total 9 days; 76 µmol/L (129.44 µg/dL)	100%
**Kosho et al**. **2000, case report**	(1) 1 day	CPS deficiency (1) with peak hyperammonemia 1790 µmol/L (1,277.9 µmol/L)	CHDF 19 days; < 200 µg/dL	100%

**Kaneko et al**. **2013, case report**	(1) 1 day	Extremely low birthweight infant with hyperammonemia to 1640 μg/dL (962.95 μmol/L)	CHD 48 h; 320 μg/dL (187.9 μmol/L) and down trending	100%
**McBryde et al**. **2006, retrospective study**	(21) 56.2 +/- 71.0 months	UCD (14), organic acidemias (5), idiopathic hyperammonemia (1), Reye syndrome (1); 721.4±467.2 μmol/L (1010.5±654,4 μg/dL	HD, CVVHD 6.1 ± 9.8 days < 200 μmol/L (280.1 μg/dL)	100%
**Gander et al**. **2017, case report**	(1) 5 days	PA with hyperammonemia at ammonia level of 1,730 μmol/L (2,946.36 μg/dL).	VV ECMO Not defined; 100 mL/h; <200 μmol/L (280.1 μg/dL)	100%
**Wen et al**. **2016, case report**	(2) 5 days	PA with hyperammonemia at ammonia level >968 μmol/L (1355.9 μg/dL)(2)	CVVHD with ECMO 33 h-HF 1000; 70-80 mL/h, 40-90 mL/h ; 142.9 μg/dL (102 mmol/L)	100%
**Summar et al**. **1996, case report**	(2) 5 days; 4 days	ASL deficiency with ammonia level at 780 μmol/L (1,328.42 μg/dL); UCD with peak ammonia level at 1500 μmol/L (2,554.65 μg/dL)	ECMO-HD 2h 24 μmol/L (40.88 μg/dL); 35 μmol/L(59.61 μg/dL)	100%
**Robinson et al**. **2018, retrospective analysis**	(13) 38.1 (IQR 37.0-39.0) weeks	ASL (6) CPS1 (5), OTC (2), organic acidemia (2), isovaleric acid CoA dehydrogenase deficiency (1), and propionyl CoA carboxylase deficiency (1). Peak ammonia level of 1041 μmol/L (1,772.93 μg/dL)	ECMO-HD 7.3h (IQR 3.6-13.5h); <200 μmol/L (280.1 μg/dL)	100%

CVVHDF, continuous venovenous hemodiafiltration; CVVHD, continuous venovenous hemodialysis; VV ECMO, venovenous extracorporeal membrane oxygenation; HD, hemodialysis; HF, hemofiltration; PD, peritoneal dialysis; CHD, continuous hemodialysis; CVVH, continuous venovenous hemofiltration; CAVHDF, continuous arteriovenous hemodiafiltration; CAVHF, continuous arteriovenous hemofiltration; CPS, carbamoyl phosphate synthetase; MMA, methyl malonic acidemia; OTC, ornithine transcarbamylase; MSUD, maple syrup urine disease; UCD, urea cycle disorders; PA, propionic acidemia; HUS, hemolytic uremic syndrome.

Supplementary Information 3: KRT protocols for managing hyperammonemia

Peritoneal dialysis (PD) protocol

1. Placement of catheter
    a. Bedside placement by abdomen puncture
        i. Establish IV access and ensure empty bladder, in order to avoid puncture of bladder wall with catheter.
        ii. Insert canula (14G or 16G) into peritoneal cavity.
        iii. Prime abdomen with 10–30 ml/kg of dialysate (skip if patient has significant ascites).
        iv. Create small needle-sized skin insertion site (to avoid incisions at insertion site and prevent leakage) and insert catheter.
        v. Connect catheter to dialysate fluid at one end and outflow drain at other end.
        vi. Run dialysate fluid to check catheter patency and observe effluent for blood or feces.
    b. Surgical placement
        i. Gold standard if a trained surgeon is available and the patient's condition permits.
        ii. Enables more-efficacious sealing of the abdomen with reduced incidence of peritoneal fluid leakage
        iii. Cuff(s) fixation enables stable installment and reduced exposure to infection risk.
        iv. In the event of delay, then a bedside PD catheter should be inserted.
2. Catheter types
    a. Neonates:
        i. Acute PD multipurpose catheters (such as Cook PD multipurpose drainage or 'pig tail' catheters 8.5F, 8cm)
        ii. In absence, use 1 or 2 large vascular catheters (e.g. 18 G) or central lines (large bore 16F) as 'improvised' catheter.
    b. In larger children:

    i. Acute PD set 9F (15cm Cook Medical Europe Ltd)
    ii. Tenckhoff straight pediatric set (37cm, Cook Medical Europe Ltd)
    iii. Tenckhoff straight neonatal/pediatric catheter (31cm, Argyle, Medtronic/Covidien)
    iv. Spiral acute PD catheter set (35cm or 39cm, Cook Medical Europe Ltd)

3. PD solutions (see Table for examples of suitable PD solutions)
    a. Avoid PD solutions containing lactate (patients may have hepatic dysfunction, primary hyperlactatemia or increased lactic acidosis)
    b. PD solutions containing bicarbonate should be used if available
    c. Additives in PD fluid:
        i. Heparin: 500–1,000 U/L for initial 24h (continue if fluid is hemorrhagic).
        ii. Potassium: 4 mEq/L if K^+<3.5 mEq/L; unless concerned about sudden rise in serum potassium.
        iii. Antibiotics: if evidence of contamination or peritonitis is present (>100 cells/mm^3, with >50% neutrophils).

Examples of suitable PD solutions

**Trade name**	**Constituents**						
	**Glucose (g%)**	**Bicarbonate (mmol/L)**	**Lactate (mmol/L)**	**Na (mmol/L)**	**Ca (mmol/L)**	**Mg (mmol/L)**	**Cl (mmol/L)**
Baxter Healthcare S.A. Castlebar, Ireland							
Physioneal 35	1.36, 2.27 or 3.86	25	10	132	1.75	0.25	101
Physioneal 40	1.36, 2.27 or 3.86	25	15	132	1.25	0.25	95
Fixioneal 35	1.36, 2.27 or 3.86	25	10	132	1.75	0.25	101
Fixioneal 40	1.36, 2.27 or 3.86	25	15	132	1.25	0.25	95
Fresenius Medical Care GMBH St. Wendel, Germany							
BicaVera 1.5	1.5	34	-	134	1.75	0.5	104.5
BicaVera 2.3	2.3	34	-	134	1.75	0.5	104.5
BicaVera 4.25	4.25	34	-	134	1.75	0.5	104.5

4. Regular PD prescription
    a. Infusion volume: initiate with 10 ml/kg body weight, gradually increase to 20–40 ml/kg body weight after verifying absence of leakage.
    b. Fill time: 5–10 min
    c. Dwell time: 30–60 min (15–20 min in neonates and infants)
    d. Drain time: 10 min
    e. Caveats:
        i. Strict control of fluid balance: eventual unwanted negative fluid balance must be monitored and replaced.
        ii. Plasma lactate levels: if PD solution with lactate is used, plasma lactate levels should be monitored.
        iii. Acid-base balance: base concentration of PD solution is appropriate for uremic patients, but alkalosis must be monitored and corrected accordingly.
    f. Manual PD
        i. Neonates: available cyclers do not allow for small volumes and there is a lack of adaptation time for low catheter performance or malfunction.
        ii. May be performed using a set created for continuous ambulatory peritoneal dialysis (CAPD; e.g. Stay-safe, Fresenius) to precisely control fluid volume in and out of the abdomen
        Automated PD: useful in larger children and provides more precision (such as Sleep-safe, Fresenius Medical Care; Homechoice Claria, Baxter Int.)

Hemodialysis (HD) protocol

1. Acquire central vascular access
    a) Newborns:
        i. 7F or 8F dual lumen catheter in internal jugular (IJ) or femoral vein
        ii. 5.0–8.5F umbilical venous catheter (UVC) for blood return, but arterial access cannot be accessed by either umbilical arterial catheters (UACs) or UVCs

        iii. 7F or 8F dual lumen catheter in the subclavian vein

    a) Older children/adolescents: pick a catheter appropriate for their body size.

2. Pick the correct dialyzer for patient weight.
    a) Neonates <3 kg: use F4 High Performance Steam (HPS) dialyzer (Fresenius)
    b) Neonates >3kg: use F6 HPS dialyzer (Fresenius)
    c) For older children: use the largest possible dialyzer
3. Prime the circuit with dilute blood in all patients <15 kg or if hemodynamically unstable
    a) Obtain a unit of diluted packed red blood cells (PRBC; hematocrit 35%).
    b) Use heparinized normal saline to prime the circuit then re-prime with non-heparinized normal saline.
    c) Use the diluted PRBC to prime the dialysis circuit.
    d) The blood is warmed by recirculation for 5 min.
4. Add 4 mEq/L KCl to the dialysate (to avoid hypokalemia in patients with normal renal function).
5. Infuse phosphate at 1–2 mg/kg/h
    a) Increase IV phosphate if serum PO_4 <3.5 mg/dL or continues to fall.
6. Dialysate flow rate (Qd) depends on age:
    a) Neonates: 500 mL/min
    b) Older children/adolescents: 800 mL/min
7. Blood flow rate (Qb) 30–50 ml/min (10–20 mL/kg/min in infants) or the maximum blood flow of the catheter in older children.
8. If serum osmolality >300 mOsm or signs of cerebral edema, administer IV mannitol (0.25 g/kg) at the beginning of dialysis.
9. Dialysate temperature 36.5^{o}C to avoid hypothermia in neonates and infants.
10. In patients <15kg or hemodynamically unstable, simultaneous access can be obtained by connecting the arterial and venous lines.
11. Preparing heparin infusion:
    a) Order heparin infusion in 10 mL syringe with 100 units/mL (<10 kg) or 1,000 units/mL (>10 kg).
    b) Check activated clotting time (ACT) promptly after beginning dialysis.

c) If ACT <200s at onset, administer an IV heparin bolus 25–50 units/kg (max 2,000 units).

d) Begin continuous heparin infusion at a rate of 10–25 units/kg/h.

e) Check ACT every 15–30 min and adjust heparin infusion according to ACT guidelines, to achieve ACTs 180–220.

f) Maintain ACT 180–220s (see Table of ACT guidelines, below)

ACT Guidelines

**ACT (s)**	**Action**
<160	Heparin bolus 10 units/kg via circuit<br>Increase heparin drip by 20%
160–180	Heparin bolus 5 units/kg via circuit<br>Increase heparin drip by 10%
180–220	No action
220–260	Decrease heparin drip by 10%
>260	Decrease heparin drip by 20%

12. Measure serum ammonia, osmolality, renal function panel and magnesium prior to start of dialysis.
13. Check serum osmolality, electrolytes, calcium, magnesium and phosphorus every 2h.
14. Check ammonia levels every 1h in case of rebound effect.
15. Consider starting continuous renal replacement therapy (CKRT) to aid rebound effect.
16. Discontinue HD treatment once serum ammonia concentrations are <100 μmol/L for two separate measurements. Patient can be switched to step-down CKRT to prevent rebound hyperammonemia. Discuss with metabolism specialist, as therapy with nitrogen-scavenging agents may be sufficient to prevent rebound.
17. When withdrawing HD, if the circuit was primed with blood then clamp the lines at completion of treatment and discard the entire apparatus containing the blood (i.e. do NOT return blood in the system to the patient to avoid acutely increasing the patient’s blood volume).

18. When HD/CKRT is stopped, consider restarting nitrogen-scavenging medications (e.g. sodium benzoate or sodium phenylacetate with arginine HCl) per metabolism recommendations. This medication should already have been initiated for hyperammonemia before dialysis for patients with UCD or be available at patient's bedside at start of HD/CKRT.

Continuous kidney replacement therapy (CKRT) protocol

1. Step-down CKRT therapy is initiated after HD to prevent rebound of ammonia >200 μmol/L by transitioning to CKRT.
2. Complete HD protocol as instructed.
3. Set up one of the following continuous detoxification methods: continuous arteriovenous hemofiltration (CAVHF), continuous arteriovenous hemodialysis (CAVHD), continuous arteriovenous hemoperfusion (CAVHP), continuous venovenous hemofiltration (CVVHF), continuous venovenous hemodialysis (CVVHD), continuous venovenous hemoperfusion (CVVHP).
4. Blood flow rates (Qb): maintain at 5mL/min/kg body weight.
5. Ultrafiltration rate: slowly increase by 0.5–2.0 mL/kg/h until fluid balance is stable.
6. Replacement and/or dialysate flow rate will total at least 2,500–3,000 mL/h/1.73m^2 up to 8,000 ml/h/1.73m^2.
7. Heparin anticoagulation preparation:
   a) Order heparin infusion in 10 mL syringe with 100 units/mL (<10 kg) or 1,000 units/mL (>10 kg).
   b) Check activated clotting time (ACT) promptly after beginning dialysis.
   c) If ACT <200, administer an IV heparin bolus 20–50 units/kg (max 2,000 units).
   d) Begin continuous heparin infusion at a rate of 10–25 units/kg/h.
   e) Check ACT every 15–30 min and adjust heparin infusion according to ACT guidelines, to maintain ACTs 180–220.
   f) Hold heparin if: activated partial thromboplastin time (aPTT) >75s, international normalized ratio (INR) >2, ACT >275s, platelet count <50,000/μL or symptoms of bleeding.
   g) Maintain ACTs 180–220s (see Table of ACT guidelines, above)

8. Check serum osmolality, electrolytes, calcium, magnesium, phosphorus, and ammonia every 2h.
9. CKRT can continue for at least 4h and/or until serum ammonia level is <100μmol/L for two separate measurements.
10. Once treatment is withdrawn, recheck electrolyte, blood gas and ammonia levels.
11. When HD/CKRT is stopped, consider restarting nitrogen-scavenging medications (e.g. sodium benzoate or sodium phenylacetate with arginine HCl) per metabolism recommendations. This medication should already have been initiated for hyperammonemia before dialysis for patients with UCD or be available at patient's bedside at start of HD/CKRT.

High-dose CKRT protocol

1. Acquire central vascular access with proper catheter size for each patient weight.
    a) Newborns: 7F dual or triple lumen catheter in IJ or external jugular (EJ) or femoral vein
    b) 3–6 kg: 7F dual lumen catheter in IJ, EJ or femoral vein
    c) 6–10 kg: 8F dual lumen catheter in IJ, EJ, subclavian or femoral vein
    d) >10–20 kg: 9F dual lumen catheter in IJ, EJ, subclavian or femoral vein
    e) >20–30 kg: 10F dual lumen catheter in IJ, EJ, subclavian or femoral vein
    f) >30kg: 12F dual lumen catheter in IJ, EJ, subclavian or femoral vein
2. Prime the circuit with PRBC and bicarbonate to help prevent bradykinin release syndrome. Make sure patient has a normal ionized calcium level.
    a) Prime PRBC tubing with 70 mL of PRBCs.
    b) Prime separate tubing with 70 mL of sodium bicarbonate.
    c) Using a Y-shaped connector, connect PRBC tubing and sodium bicarbonate tubing to the central vascular access line.
    d) Set dialysate at prescribed rate.
    e) Connect CKRT circuit to sterile saline bag.
    f) Start CKRT machine with blood flow at 20 mL/min, PRBC at 10ml/min and sodium bicarbonate at 10ml/min for about 5–6 min.
    g) Start calcium gluconate infusion via central line.

h) Start anticoagulation at prescribed rates.

i) After blood prime is complete, stop CKRT machine and PRBC and sodium bicarbonate infusions.

j) Disconnect CKRT from sterile saline bag and connect to the venous line.

k) Restart CKRT to prescribed rate.

3. Blood flow rate (Qb): 30–50 mL/min.
4. High-dose dialysate flow rate (Qd): 1,000 mL/h (8,000 mL/1.73m^2/h)
5. Dialysate/replacement fluid should contain 4 mEq/L of potassium and 2 mEq/L of phosphate.
6. If utilizing heparin anticoagulation:

a) Order heparin infusion in 10 mL syringe with 100 units/mL (<10 kg) or 1,000 units/mL (>10 kg).

b) Check ACT promptly after beginning dialysis.

c) If ACT <200s, administer an IV heparin bolus 20–50 units/kg (max 2,000 units).

d) Begin continuous heparin infusion at a rate of 10–25 units/kg/h.

e) Check ACT every 15–30 min and adjust heparin infusion according to ACT guidelines to maintain ACTs 180–220s.

f) Maintain ACT 180–220s (see Table of ACT guidelines, above)

7. If utilizing citrate anticoagulation:

a) Infuse citrate into blood at the beginning of the CKRT circuit dosed at 0.75mL/h times the blood flow in mL/min (half the usual rate).

b) 0.5% citrate solution with 140 mEq/L sodium concentration serves as both anticoagulation and replacement solution. Maintain bicarbonate levels within solution at 22–25 mEq/L.

    1. Administer solution to the arterial access starting at 1,000–1,500 mL/h with a blood flow rate of 150–200 mL/min.
    2. Monitor calcium concentrations and maintain ionized Ca concentration in the circuit <0.35 mmol/L, correlating to a citrate blood concentration of 4–6 mmol/L.

8. Monitor patient’s electrolytes, platelets, ammonia levels, magnesium and ionized calcium (espescially when using citrate anticoagulation) every 1–2h.
9. Continue CKRT treatment until ammonia level is <100 μmol/L.

10. Once treatment is withdrawn, recheck electrolyte, blood gas and ammonia levels.
11. When HD/CKRT is stopped, consider restarting nitrogen-scavenging medications (e.g. sodium benzoate or sodium phenylacetate with arginine HCl) per metabolism recommendations. This medication should already have been initiated for hyperammonemia before dialysis for patients with UCD or be available at patient's bedside at start of HD/CKRT.
